# Teeth of Gold

**Published:** 1878-11

**Authors:** 


### TEETH OF GOLD.

A new method of adding artificial crowns to the roots of teeth is now being introduced to the profession by Dr. C. M. Richmond. It consists in making the crowns for the molars and bi-cuspids wholly of gold, twenty to twenty two carets fine, of the size and form of the natural teeth—with the prominces as in nature—these are attached to the roots by a band of gold perfectly fitted round the necks of the roots at and beyond the margin of the gum. Os artificial is used for filling any space that might exist after the crown is placed on the roots. This mode, if it proves successful, renders the roots of molars and bi-cuspids, which have usually been regarded as of no value, at least for the support of artificial crowns, very important.

For the anterior teeth an ordinary plate tooth is used, and with gold in connection with this, the attachment is made on the same principle as that described for the molars and bi-cuspids, thus giving the porcelain front, and the palatine part gold. Crowns may in this way be more firmly attached than by the common method. As to durability, time must decide, though the method has been in use two or three years in over two hundred cases, and as yet a very small per cent of failures has been reported. As in almost every other operation on the teeth, permanence will depend much upon the case, together with the manner in which the work is done. It is a method certainly worthy the attention of all who desire to do the best for the unfortunate committed to their care.

